# Advanced Situation with Recombinant Toxins: Diversity, Production and Application Purposes

**DOI:** 10.3390/ijms24054630

**Published:** 2023-02-27

**Authors:** Elena Efremenko, Aysel Aslanli, Ilya Lyagin

**Affiliations:** Faculty of Chemistry, Lomonosov Moscow State University, Lenin Hills 1/3, Moscow 119991, Russia

**Keywords:** recombinant toxin, protein, exotoxin, killer toxin, prion, antivenom, antidote, toxicity prediction, detoxification, enzyme

## Abstract

Today, the production and use of various samples of recombinant protein/polypeptide toxins is known and is actively developing. This review presents state-of-the-art in research and development of such toxins and their mechanisms of action and useful properties that have allowed them to be implemented into practice to treat various medical conditions (including oncology and chronic inflammation applications) and diseases, as well as to identify novel compounds and to detoxify them by diverse approaches (including enzyme antidotes). Special attention is given to the problems and possibilities of the toxicity control of the obtained recombinant proteins. The recombinant prions are discussed in the frame of their possible detoxification by enzymes. The review discusses the feasibility of obtaining recombinant variants of toxins in the form of protein molecules modified with fluorescent proteins, affine sequences and genetic mutations, allowing us to investigate the mechanisms of toxins’ bindings to their natural receptors.

## 1. Introduction

**Figure 1:**
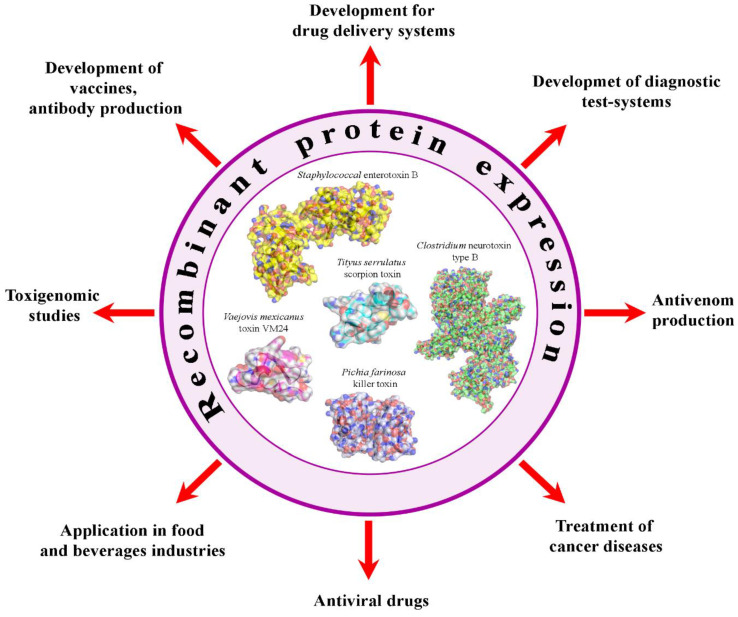
Various applications of recombinant toxins.

To date, recombinant toxins from various biological sources (bacteria, yeast, scorpions, snakes, spiders and other living organisms) are widely used as: (i) antimicrobial agents for medical purposes, as well as antimicrobial additives for the food and biotechnological industries, (ii) groundwork for the creation of drugs with anticancer activity and the treatment of neurodegenerative diseases and (iii) the basis to develop vaccines, etc. Multiple works have been performed to study the mechanisms of action of genetically modified toxins and their applications [1,2,3,4,5,6] (Figure 1).

The protein/polypeptide nature of most of these natural toxins allows them to obtain their recombinant forms. The potential for developing these biomolecules in high enough quantities is the basis for further advancements in developing vaccines and drugs with reduced cost and their widespread use, on the one hand. On the other hand, the production of recombinant toxins avoids the need to work directly with the natural sources of these biomolecules (animals and microbial pathogens). Obtaining genetic constructs encoding the synthesis of recombinant toxins expands the possibilities of their synthesis in special modified forms. Like many recombinant proteins, recombinant toxins can be obtained in high yields using different expression systems, including extracellular secretion, and further isolated and finely purified using affine carriers [7,8].

Various modifications, which in this case can be introduced into such recombinant proteins, can lead to a weakening of the toxic potency of the resulting toxins or, conversely, increase their toxicity. Thus, it is important to publicly discuss the situations and monitor the emergence of such developments. Specifically, it is possible to preliminarily assess the toxicity of the resulting proteins using bioinformatics tools and databases. The issues of the targeted production of recombinant proteins with toxic activities for a variety of uses have been raised multiple times in various reviews in recent years [9,10]. However, each of these scientific analytical publications was focused on discussing specific and rather narrow areas in ongoing research.

This review is aimed at a broad analysis of a currently known variety of recombinant toxins, their systematization according to both their origins and purposes of their production and the possibility of predicting the level of toxicity of new variants of recombinant toxins based on the current level of computer science development in analysis of protein structures. Special attention in the review was focused on developments aimed at finding enzymatic solutions (i.e., antidotes) for detoxification of individual recombinant toxic proteins. It was interesting from a scientific and practical standpoint to determine new achievements and main trends during last 5 years in this field, including results obtained by researchers in Russia and worldwide.

## 2. Spectrum of Recombinant Toxins and Their Origins

Toxins produced by various living organisms are the main pathogenic factors causing severe diseases and poisoning of humans and animals. At the same time, one organism can synthesize a number of different toxins responsible for multiple symptoms and effects of intoxication.

Most of proteinaceous toxins well-studied to date are produced by various bacteria. However, toxins that are found in yeast, snake, scorpion and spider venoms and other living organisms are also actively studied by various scientific groups today. Recombinant toxins obtained from various origins and purposes of their obtaining are presented in Table 1 [11,12,13,14,15,16,17,18,19,20,21,22,23,24,25,26,27,28,29,30,31,32,33,34,35,36,37,38,39,40,41,42,43,44,45,46,47,48,49,50,51,52,53,54,55,56,57,58,59,60,61,62,63,64,65,66].

### 2.1. Bacterial Recombinant Toxins

Bacterial cells are capable of synthesizing endo- and exotoxins. Endotoxins, as a rule, are cell-bound lipopolysaccharides that are released after cell destruction, while exotoxins are protein toxins that are synthesized inside cells and released into the environment. Thus, the recombinant forms of these exotoxins are discussed next (Table 1).

Botulinum neurotoxin (BoNT) and tetanus toxin (TeNT) produced by cells of the *Clostridium botulinum* and *C. tetani*, respectively, are among the most dangerous and therefore the most well-studied bacterial toxins. Botulism and tetanus diseases caused by these toxins are among the most severe neurological diseases that cause flaccid paralysis and spastic paralysis, respectively. In addition, BoNT is widely used to treat a number of diseases. Consequently, recombinant forms of these toxins have been actively created and researched for many years with the aim of both developing effective antidotes and obtaining drugs based on them.

A double-blind, placebo-controlled study evaluated the safety, tolerability and pharmacodynamics (PD) of the recombinant botulinum toxin serotype E (rBoNT-E) compared with commercial botulinum toxin type A (ABO, Dysport ^®^) [11]. All doses of the recombinant toxin were well tolerated, and rBoNT-E had a faster onset of action, a greater peak effect and a shorter duration of effect at the highest tested doses compared with ABO.

To solve an opposite task and neutralize BoNT and other toxins, various antibodies are usually used. Special interest is afforded to single-domain camel antibodies (sdAb, VHH or nanobody) possessing unique structure and characteristics and their chimeras with usual human immunoglobulins. As a result, such immunotherapeutic agents could have up to 1000 times increased protective activity against *C. botulinum* and prolonged circulation in blood [47].

Different subtypes of BoNT have a varying toxicity, and BoNT/A is more potent toward the human neuroblastoma cell line as compared to BoNT/B [53]. At the same time, genetic modification of the latter to BoNT/BY resulted in improved affinity for human synaptotagmin and BoNT/B receptor, as well as increased toxicity toward this cell line.

C3 protein toxin from *C. botulinum* (C3bot) cells is a mono-ADP-ribosyltransferase that selectively intoxicates macrophages, osteoclasts and dendritic cells by cytosolic modification of Rho GTPases (Rho-A, Rho-B and Rho-C). Thus, C3bot and, even better, its nontoxic variant C3bot_E174Q_ have been proven as perspective transporters for selective delivery of small molecules, peptides and proteins to the cytosol of macrophages and other cells [25,26].

Proteolytically activated separate binding/transport subunit C2IIa of C2 toxin from *C. botulinum* has been found [27] to be a specific inhibitor of chemotaxis of polymorphonuclear neutrophils (PMN), allowing selective suppression of excessive and harmful PMN recruitment to organs as a result of trauma. The enzymatically inactive N-terminal part of the *C. botulinum* C2 toxin (C2IN) when fused to Rho-inhibiting C3 toxin from *C. limosum* (C3lim) significantly improves the toxic action of the latter [54]. In a clinically significant mouse model, the in vivo introduction of C2IN-C3lim into the lungs after a blunt chest injury prevented injury-induced recruitment of monocytes into the lungs. Thus, such combinatorial fusion chimeras can be of practical interest due to great variability of available toxin modules.

Until now, vaccination has been the best way to combat diseases associated with many bacterial strains, including *C. perfringens* cells and α-, β- and *ε*-toxins of the bacteria. However, commercially available vaccines are based on inactivated toxins and have many manufacturing disadvantages that can be overcome using recombinant antigens. Recombinant α-, β- and ε-toxins were synthesized in *E. coli* cells to create a trivalent vaccine and evaluated on rabbits, cattle, sheep and goats. The levels of produced antibodies in all animals exceeded the minimum values recommended by international protocols [48,49], thus proving the viability of the approach. Even more, nonvirulent species of the same bacteria can be modified to bear a specific toxin or its part and safely modulate strong immune response, e.g., *Vibrio cholerae* cells expressing the β-subunit of cholera toxin (CTB) [50].

Another major group of proteinaceous toxins is produced by the members of the genus *Bacillus*. *Bacillus cereus* cells causing foodborne diseases secrete various pore-forming pathogenicity factors, including Hemolysin II (HlyII). As above mentioned, it can be specifically neutralized by antibodies [29], thus preventing mortality in vivo [30].

*B. anthracis* cells cause one of the most dangerous infectious diseases, Anthrax. The use of the Anthrax protective antigen (PA) is considered the most promising approach to the development of an Anthrax vaccine. However, the instability of the recombinant PA complicates the production of stable recombinant vaccines. Thus, a number of modification methods have been applied in recent years to design a stable recombinant Anthrax PA. For example, proteolytic-sensitive sites simultaneously with deamidation-prone amino acids can be genetically modified [58,59]. Alternatively, additional stabilizers, e.g., spherical particles (SPs) of tobacco mosaic virus, can be added [60]. Joint application of both methods gives even better results in terms of stability, immunogenicity and protectiveness of the final product, including in vivo tests with a fully virulent *B. anthracis* strain.

*B. thuringiensis* cells produce δ-endotoxins (Cry), which are toxic to a wide variety of insect pests and currently used widely in agriculture. Insertion of the gene encoding Cry1Ia toxin into a bacterial strain inhibiting fungal growth results in combined fungistatic and insecticidal activity as well as ability to induce plant resistance [31].

A lot of bacterial toxins in their structures contain metal ions performing various purposes. First of all, metal ions can be located in the active sites of metalloproteases such as BFT toxin from *B. fragilis* leading to damage and necrosis of the intestinal epithelium [32]. In addition, such metals can contribute to toxin structural stabilization and even promote recognition of the target receptor like in the case of staphylococcal enterotoxin-like protein P (SElP) from *Staphylococcus aureus* binding to major histocompatibility complex class II (MHCII) [61]. It should be noted that some virulence factors secreted by bacteria may be toxic to the microorganisms themselves. To prevent collateral damage and to additionally protect active components, they can be secreted in nanovesicles, which are able to be modeled in silico [32].

The diphtheria toxin (DT) from *Corynebacterium diphtheriae* kills mammalian cells by inactivating the elongation factor EF-2. The translocation domain in DT plays a critical role in allowing the catalytic domain to pass to the cytosol from endosomal compartments and can be used as a functional vector for active transport of protein drugs [33].

Some mammalian species are resistant to DT. The DT receptor, proHB-EGF, in resistant and sensitive species differs by amino acid sequence and therefore by secondary structure; however, there is no consensual opinion on how the difference in the structure of primary receptors changes the process of internalization of DT by resistant cells compared to sensitive ones. According to some publications [34], there can be even very little difference of binding constants of DT subunit B (which includes receptor-binding and translocation domains) to resistant and sensitive cells, while there was huge difference of intracellular concentrations of toxin within model cells. It means that multiple mechanisms of resistance to DT may exist in mammalian cells.

Several approved drugs, e.g., denileukin diftitox, which is fusion of DT with interleukin-2, are commercially available and actively used to date. However, research to improve their efficiency, producibility and safety as well as to obtain new therapeutics with DT are constantly continued [56,57].

*Listeria monocytogenes* cells apply internalins InlA and InlB to attach and penetrate into mammalian cells. Curiously, hepatocyte growth factor receptors (HGFR) together with other multiple variants are also affected by InlB [35]. This is important since HGF/HGFR play crucial role in liver restoration after its acute toxic damage. Thus, truncated bacterial InlB was implemented as a functional analogue of HGF to obtain novel drugs with hepatoprotective activity.

Bacterial toxins can interact not only with receptors themselves but with complexes of receptor and signal molecules. One of such examples is LcrV from *Yersinia pestis* [36]. It is a strong virulence factor having multiple functionalities, one of which is specific activation of human receptor-bound interferon-γ (hIFN-γ), which resulted in immune cell death via apoptosis. It became possible only after hIFN-γ binding to receptor and presentation of its ^138^GRRA^141^ site, which specifically interacts with ^32^LEEL^35^ and/or ^203^DEEI^206^ sites of LcrV. Thus, inactivation of these sites by specific antibodies completely prevents any harmful effects of LcrV.

Protein biosynthesis can be targeted by bacterial toxins, as well. For example, bacteria can utilize multiple enzymes from Gcn5-related N-acetyltransferase (GNAT) superfamily to acetylate and thus inactivate specific aminoacyl tRNAs, including transporters of Met, Ile, Gly, etc. [37].

### 2.2. Yeast Recombinant Toxins

Killer yeasts are able to produce proteins named “killer toxins” that are often glycosylated and bind to specific receptors on the surface of the sensitive microorganism, which is then destroyed by a target-specific mechanism of action (Table 1). They are widespread among yeasts and attract a lot of attention of researchers. To date, more than 100 types of killer yeasts have been described [67]. The most well-characterized killer toxins in terms of their genetic determinants, biochemical characteristics, molecular targets on sensitive cells and mechanisms of their destruction are toxins K1, K2 and K28 from *Saccharomyces cerevisiae*; zymocin from *Kluyveromyces lactis*; PMKT and PMKT2 from *Pichia membranifaciens*; PaKT from *Wickerhamomyces anomalus*; HM-1 from *Cyberlindnera mrakii* and Kpkt from *Tetrapisispora phaffii*. Due to their properties and spectrum of action, which is aimed at pathogenic microorganisms, recombinant killer toxins are being actively investigated in order to develop therapeutic agents based on them. However, the lack of research on their effects on humans and animals limits their use in the food and feed industry. Another drawback is that additional information about the mechanisms underlying the formation of killer toxins in yeast is required. Without solving these issues, it is not possible to successfully implement killer toxins in practice [67,68].

A study of *S. paradoxus* revealed a new K1-like toxin (K1L) being active against sensitive competing yeast cells [12]. It is encoded by double-stranded RNA (dsRNA) and satellite dsRNA, which may also be of virus origin. Its homologues have been identified in other six yeast species not belonging to *Saccharomyces* and are likely to be acquired by horizontal gene transfer via dsRNA and/or DNA with subsequent diversification of their structure and toxicity profile.

Genetic fusion of toxins with fluorescent proteins allowed researchers to study the binding of the toxin to the cell envelope of affected yeast [13]. However, intracellular translocation of labeled recombinant toxin K28 was not observed then, in spite of the presence of toxicity. It means there are gaps in our understanding of the true mechanism of killer toxin action and transport even among best-investigated ones. Further research is required to visualize intracellular transfer of toxins using high-resolution imaging techniques of individual molecules.

Killer toxin K1 is secreted by *S. cerevisiae* strains in a heterodimeric form. After binding to the primary receptor (β-1,6 glucans) in the cell wall, K1 is transported to the plasma membrane and is initially supposed to interact with its secondary receptor Kre1p, which ultimately leads to an ionophoric disruption of the membrane function. However, expression of recombinant K1α in resistant yeasts lacking Kre1p resulted in profound toxic effect [14], thus excluding role of the receptor. At the same time, co-expression of toxin precursor(s) in sensitive cells eliminated any negative effects. Thus, resistance to killer toxins is a part of adaptive (acquired) immune system.

Some killer toxins, e.g., Kpkt from *T. phaffii* (formerly *Kluyveromyces phaffii*), have antimicrobial activity not only on yeast but also on bacteria [15]. Interestingly, activity of Kpkt was not detected toward all tested mycelial fungi. Meanwhile, Kpkt has a β-1,3-glucanase activity [16,38] and thus can be combined, for example, with chitinases to synergistically improve their antifungal effects. At concentrations effective again yeasts, recombinant Kpkt has no effect on immortalized human epidermal keratinocyte cell line HaCaT [38]. That makes it promising for further investigations.

### 2.3. Recombinant Toxins of Various Animals

Venoms of snakes, scorpions and spiders are used by animals as their own defensive and offensive means by immobilizing victim and blocking the functional activity of their cardiovascular, respiratory and/or nervous systems. Proteinaceous toxins are the main components of these systems and modulate important ion channels and receptors after introduction into the body. Today, powerful databases of poisons and protein toxins with improved properties have been assembled already for more selective action, resistance to the effects of proteases, less immunogenicity and improved characteristics, in terms of pharmacokinetic properties. These characteristics can be improved by genetic modification of amino acid sequences, addition of disulfide and ion bridges, etc. After all, animal venom toxins are of great interest for applications in medicine as a basis for drug development [5] (Table 1).

The β/δ agatoxin-1 of the spider *Agelena orientalis* was obtained in recombinant form in the entomopathogenic fungus *Lecanicillium muscarium* with a special secretory signal peptide [18]. Further toxin was fused with eGFP to simplify the screening procedure. Unfortunately, toxic activity of the fusion protein was not investigated in the work.

Another fusion protein of GFP with agitoxin-2 from scorpion *Leiurus quinquestriatus hebraeus* was more useful [64]. That allowed researchers to visualize the binding of toxins to their receptor as well as to determine dissociation constants of various toxins competing for the same Kv1.3 channel.

Purotoxin-1 (PT1) from the venom of the Central Asian spider *Geolycosa* sp. selectively inhibits the purinergic receptor P2X3 and is a potent analgetic. It can be produced in pilot scale as self-cleavable fusion protein with mini-intein DnaB [19]. However, its purification is multistage and labor-intensive with modest yield at the end.

Interestingly, Tbo-IT2 toxin was identified in the spider *Tibellus oblongus* by cDNA analysis of the transcriptome of its venom glands [42]. Its amino acid sequence has a 41% identity match with the closest protein toxin, while its spatial structure folds into a well-known inhibitory cysteine knot (ICK). The first main difference is the formation of five disulfide bonds instead of the typical three that should result in extreme stability of the toxin. The second and the most puzzling difference is that Tbo-IT2 did not have inhibitory activity on the tested panel of available ion channels and neuroreceptors, while it is still toxic to the housefly, *Musca domestica*. Further research may elucidate the target(s) of the Tbo-IT2.

Another attempt to apply mini-intein DnaB was a little bit more successful [23], although the target toxin, APHC3 from the anemone *H. crispa*, which has analgesic activity, was produced in inclusion bodies and multistage purification was still required.

Fusion with His-tag and Smt3-leader peptide was shown to be a much more efficient method [24]. The resulting inhibitor of the TRPV1 ion channel (HCRG21 peptide from the sea anemone *H. magnifica*) was easier to purify and after cleavage was obtained at comparable yield to APHC3.

As stated previously with bacterial toxins, antibodies are used almost exclusively in antivenoms [22]. Combining several recombinant toxins in simple mixture [17,51] or even in fusion protein [65] often leads to improved efficiency of antivenoms, including comparing to commercial ones. Furthermore, it was found that rationally selected toxin-specific single-stranded DNA aptamers can exhibit broad cross-reactivity in vitro and ex vivo against isoforms of toxins found in various snake venoms [52].

Computer modeling provides powerful tools to thoroughly solve even complicated issues. For example, interaction of proteinaceous toxins KTx from scorpion *M. eupeus* with potassium channels (KV) was simulated and explained, followed by modulation of their activity using genetic modification [40,41]. In other work [45], authors have investigated binding of TFTs with novel receptors. Secondary structures of multiple actinoporins Hct from the sea anemone *Heteractis crispa* were generated [69], followed by analysis and successful structure–activity hypothesis testing.

Venoms contain a large number of biologically active compounds with diverse activities. Shorter peptides, e.g., azemiopsin acting on neuroreceptors [20] and bradykinin-potentiating peptides (i.e., affecting blood pressure) [21], could be prepared by a solid phase synthesis using a general Fmoc-method, while larger polypeptides are the most rational to produce using common expression systems [44,45].

A modulatory effect of some proteinaceous toxins on neuroreceptors is worth mentioning. Well-known three-finger toxins (TFTs) and their analogues [44,45] as well as azemiopsin bind mostly nicotinic acetylcholine receptors (nAChRs), but γ-aminobutyric acid receptors (GABARs) can also be affected [45].

Pore-forming toxins, e.g., Hct from the sea anemone *H. crispa* [46], have a wide nonspecific action and are almost equally cytotoxic to normal and malignant cells. However, fusing them with targeting partners, such as site-specific ligands, toxins or antibodies, could result in new drug platform development.

Recombinant toxins can be easily genetically modified and truncated to help researchers investigate their toxic action in a more detailed way. For example, peptide Ms 9a-1 from sea anemone *Metridium senile* causes significant analgesic and anti-inflammatory effects by desensitization of TRPA1-expressing sensory neurons, and it was thought to be a positive modulator of TRPA1 channel. However, truncation of its unordered domains on the N- or C-terminus resulted in complete loss of analgesic and anti-inflammatory activities in vivo [66]. Thus, another target receptor(s) is likely present in neurons.

### 2.4. Recombinant Prions

Prions (Pr) are infectious agents that cause devastating and incurable disorders known as transmissible spongiform encephalopathies (TSE). With the advent of innovative technologies, such as protein misfolding cyclic amplification (PMCA) and real-time quaking-induced conversion (RT-QuIC), in vitro amplification of prions has become possible. There is evidence suggesting that prion complexes can acquire high-order assemblies in vivo, which may look structurally ordered. However, the biophysical nature of these structures and their role in amyloid biology are still unclear. Despite the fact that the amyloid collected in vitro has some biochemical similarities with the ex vivo amyloid of the same protein, it often does not reproduce the biological activity of the latter. For example, preparations of prion protein (PrP), which are resistant to proteinase K and obtained exclusively from one recombinant PrP (rPrP), may not have any detectable infectious activity both in cell cultures and in animal bioassays. However, the proteinase K-resistant PrP obtained from rPrP is infectious if it is placed in the homogenate of a diseased brain ex vivo using the PMCA assay [70].

To study the rPrP, mechanisms of the development of toxicity and pathogenicity of prion diseases as well as their role in the development of pathologies of the nervous system is an important task of the world scientific community (Table 2, [71,72,73,74,75,76,77]).

Recent studies have shown that the infectivity of prions and their neurotoxicity may not be related to each other. Therefore, it is important to distinguish directly infective prions and those with a toxic effect, since the current hypothesis suggests that it is not the prions themselves that are toxic but another type of protein responsible for the toxicity of the disease. This species may be a by-product of prion formation, in a non-pathway amyloid PrP structure or even a non-protein whose formation is catalyzed by a prion [78]. Thus, using highly purified infectious prions, it was demonstrated that prions are not directly neurotoxic and that the toxicity presented in infected brain tissue may be different from infectious prions [76].

rPrP was obtained using the insect baculovirus cell expression system (Bac-rPrP) [71] to determine whether pathogenic Bac-pathogenic PrP (PrP^Sc^) is produced spontaneously in intermittent ultrasound reactions. No spontaneous formation of Bac-rPrP^Sc^ was observed at 37 °C, but when the reaction temperature increased to 45 °C, Bac-rPrP^Sc^ was formed in all samples studied. Some variants of Bac-rPrP^Sc^ were transmitted to mice, but when the reaction was repeated for 40 cycles, transmissibility was lost. It is noteworthy that various variants of Bac-rPrP^Sc^, including nontransmissive ones, were characterized by resistance to proteinase K and were dependent on the presence of cofactors during amplification. However, their characteristics also disappeared after 40 reaction cycles, and the variety converged on one variant. These results show that different variants of Bac-rPrP^Sc^ are generated with different transmissivity to mice and structural properties; variants of Bac-rPrP^Sc^ compete with each other and gradually converge to a variant with a slightly higher amplification rate.

To understand the role of the hydrophobic region in the formation of an infectious prion at the molecular level, X-rays of crystal structures of mouse PrP (MoPrP, residues 89–230) in complex with a nanobody (Nb484) were obtained [72]. Using a rPrP reproduction system, it has been shown that binding of Nb484 to the hydrophobic region of MoPrP effectively inhibits the reproduction of proteinase-resistant PrP^Sc^ and the infectivity of prions. In addition, when added to cultured mouse brain slices in high concentrations, Nb484 did not exhibit neurotoxicity, which is sharply different from other neurotoxic antibodies against PrP. Thus, Nb484 may be a potential therapeutic agent against prion disease.

Five groups of transgenic mice expressing elk PrP (TgElk) were vaccinated with either one CpG adjuvant or one of four rPrP immunogens: deer dimer (Ddi); deer monomer (Dmo); mouse dimer (Mdi) and mouse monomer (Mmo) [73]. Then mice were intraperitoneally infected with prions of chronic wasting disease (CWD). All vaccinated mice developed anti-PrP antibody titers detected by ELISA. It is important to note that all four vaccinated groups survived longer than the control group, while in the group immunized with Mmo, the average survival time increased by 60% compared to the control group (183 vs. 114 days after inoculation).

Thus, the use of recombinant forms of prions allows researchers to study their immunogenicity and to develop novel vaccines.

In order to establish how various cofactors modulate the formation and selection of prion strains, PMCA was used to generate a variety of infectious rPrP strains by multiplication in the presence of brain homogenate [77]. It is known that brain homogenate contains certain cofactors whose identity is only partially known and which facilitate the transformation of normal PrP (PrP^C^) into PrP^Sc^. A mixture of various infectious prion strains was obtained and introduced into the brain homogenate, where various polyanionic cofactors were present. These cofactors could control the evolution of mixed prion populations toward the development of specific strains (types of conformations). As a result, it has been shown that various infectious rPrP can be obtained in vitro. Their specific conformation (strain) depends on the cofactors available during reproduction. 

These observations are very important for understanding the pathogenesis of prion diseases and their ability to reproduce in various tissues and hosts.

The RT-QuIC method can be used to detect pathogenic PrP in various biological tissues of humans and animals. However, this method requires a continuous supply of freshly purified PrP and thus is not available in a diagnostic laboratory. To solve the issue, a method for obtaining a rPrP has been developed [74]. Lyophilized rPrP from bank vole (BV rPrP) can be stored for a long time before use, as well as be transported at certain temperatures to appropriate diagnostic laboratories, which can facilitate implementation of the RT-QuIC method as a diagnostic tool [74].

Nucleic acids have been shown in recent studies to act as potential cofactors of protein aggregation and prionogenesis. For example, RNAs, regardless of their sequence, source and size, modulate rPrP aggregation in a bimodal manner, affecting both the degree and the rate of rPrP aggregation depending on the concentration [75].

## 3. Diversity of Modern Purposes for Obtaining Recombinant Toxins

Finding ways of obtaining effective antibodies and the development of vaccines against recombinant toxins is one of the main goals today [79,80]. For maximal quality and efficiency of immunologic medications, initial toxins should be highly purified, be in sufficient quantities and stimulate selective immune response. Recombinant toxins’ production solves the first two issues, though vaccines can still have cross-specificity.

Today, different recombinant vaccines against diseases caused by pathogenic *Clostridium* genus (botulism, tetanus, black disease, etc.) are actively developed [8,9,10]. Due to the simplicity of preparation and effectiveness of action, recombinant bacterin is one of the intensively promoted for the production of immunizers against *C. perfringens* [48,49]. A number of promising experimental multivalent recombinant vaccines made up of CPA, CPB, ETX, NetB, HC-BoNT/A, HC- BoNT/B and HC-BoNT/E have been reported [10]. Recently, recombinant, genetically detoxified, full-length tetanus toxin protein (8MTT) was described and found to be an immunogenic antigen and effective as a carrier protein for peptide and polysaccharide conjugates [81]. A study on fusion toxin combining recombinant CPE (C-terminal region) from *C. perfringens* and the subunit of the Shiga toxin (*E. coli*) showed that it is effective to stimulate an immune response in mice [82]. Furthermore, monovalent recombinant vaccines Iota and TpeL have been expressed separately in *E. coli* [83]. Bioinformatics tools were also used to design new versions of recombinant CPB [48].

Anthrax toxin from *Bacillus anthracis* cells is one of the most dangerous toxins, considered among other things as a potential bioterrorism agent. In this regard, there is a huge interest in the development of vaccines based on Anthrax recombinant PA (rPA). Subunit vaccines based on rPA have a good safety and protection profile; however, rPA is unstable antigen, especially with aluminum adjuvants. The development of modern vaccines based on adjuvant compositions in the dry forms of mutant PA variants resistant to proteolysis and deamidation should help solve the problem of neutralization of Anthrax toxin. An analysis of scientific databases was carried out recently [79] with an emphasis on causes of PA instability and finding solutions to this problem. New approaches to rPA expression, new formulations of rPA-based vaccines and the simultaneous use of PA with other Anthrax antigens were reviewed in this study. A number of rPA-based vaccine candidates, including DNA vaccines and vaccines based on viral and live bacterial vectors and plant expression systems, are currently under development [79]. The protective efficacy of human sera from vaccinated individuals with a new rPA vaccine (GC1109) against Anthrax challenge has been demonstrated in passive transfer studies [84]. A DPX-rPA-based vaccine generated neutralizing antibodies titers toward Anthrax PA toxin in animal models [85]. Furthermore, modified variants of the Anthrax antigen have been obtained simultaneously through deamidation-prone asparagine residues substitution and by inactivation of proteolysis sites, and stability of modified rPA has been demonstrated under various temperature conditions [59]. Recombinant lethal Anthrax toxin (LeTx) has recently been successfully used to treat breast cancer cell line MBA-MD-231 [28].

The most recombinant vaccines are developed with a narrow spectrum of effective action. It may be a significant problem, since microorganisms secrete a lot of toxins simultaneously. Meanwhile, it is possible to create multiantigenic nanotoxoids using the natural affinity of virulence factors to cell membranes [86]. The obtained polyvalent nanotoxoids are able to deliver combination of virulence factors, are safe both in vitro and in vivo and can cause functional immunity capable of fighting live bacterial infections in model animals.

Today, protein toxins play a very important role in the treatment of advanced solid tumors by either directly destroying tumor cells or by changing the cellular processes occurring in them. Due to advancements in biomolecular methods, a number of nonpathogenic bacteria have been genetically modified for use in the development of bacterial anticancer drugs [2,87]. Immunotoxins, which consist of a protein derived from bacterial, fungal, plant toxins or human cytotoxic proteins and conjugated to a specific targeting molecule, have demonstrated high cytotoxicity to cancer cells. They are engineered to recognize disease-specific target receptors and kill the cell upon internalization. To date, a number of immunotoxins are under clinical trials, and some of them have been approved by U.S. Food and Drug Administration for the treatment of hematological tumors. However, there are some disadvantages, including immunogenicity, nonspecific toxicity and poor penetration, that need to be addressed [88]. Among the available cytolytic toxins, the *Pseudomonas* exotoxin (PE) and DT are extensively studied [89]. Their main mechanism of action is based on blocking protein synthesis via ADP-ribosylation of eukaryotic elongation factor 2. Suicide-gene therapy strategies, where controlled tumor-specific expression of DT is used for the eradication of malignant cells, are becoming increasingly important [90]. A new immunotoxin based on shortened DT389 fused to humanized scFv YP7 selective to an oncophetal marker Glypican-3 (GPC3) has recently been developed and studied against hepatocellular carcinoma (HCC) cells [55].

Recombinant immunotoxins based on PE have demonstrated significant efficacy in the treatment of tumors and autoimmune diseases. Recent strategies for structural optimization of these immunotoxins, combined with mutagenesis approaches, have reduced the side effects associated with their immunogenicity and nonspecific cytotoxicity and, as a result, led to an increase in both their safety and efficacy [91]. Recently developed immunotoxins, comprising of truncated *Pseudomonas* exotoxin A (PEA) and DT conjugated to trastuzumab, have demonstrated the potential to reduce the therapeutic dose of the trastuzumab [92]. An advanced modification technology was used to construct the site-specifically conjugated immunotoxin based on immunoglobulin G (IgG) and *Pseudomonas* exotoxin A (PE24). The constructed immunotoxin demonstrated specific toxicity toward HER2-positive cancer cell at very low concentrations [93]. The combination of two highly toxic proteins (a low immunogenic variant of PEA and ribonuclease Barnase from *B. amyloliquefaciens*) allowed the achievement of directed anticancer therapy toward HER2 and EpCAM [94]. The use of AgNPs as a delivery system for targeting of toxins to cancer cells was demonstrated for the first time by the investigation of the inhibitory effect of the nanotoxin comprised of truncated *Pseudomonas* exotoxin (PE38) loaded silver nanoparticles (AgNPs) on the viability of breast cancer cells through apoptosis [62].

The Stx toxin from Stx-producing *Shigella dysenteriae* and enterohaemorrhagic *E. coli* is another promising toxin for use as a delivery system. Engineered StxB-based drug delivery systems have the potential to deliver small or macromolecular drugs to specific intracellular organelles/cancer targets. After binding to ganglioglobotriaosylceramide (Gb3, CD77), the nontoxic subunit B (StxB) of the Shiga-holotoxin is endocytosed and delivers its payload by a unique retrograde trafficking pathway via the endoplasmic reticulum to the cytosol [95,96].

Despite the widespread use of bacterial recombinant toxins in cancer therapy, toxins from other sources are also being considered as potential anticancer agents today. Thus, a recombinant form of a new short-chain toxin isolated from the venom of the scorpion *Mesobuthus eupeus* MeICT was used to treat glioma [39]. Recombinant toxin MeICT-His in low concentration significantly inhibited the proliferation and migration of glioma cells. In vivo studies have not revealed the toxicity of MeICT when administered to mice in high doses. Studies of the effect of MeICT on the expression of mRNA MMP-2, annexin A2 and FOXM-2, which are key molecules in the development and invasion of glioma, have shown a significant decrease in the expression of mRNA annexin A2 and FOXM1.

The effect of nicotine acetylcholine receptor (nAChR) antagonists, α-conotoxins of sea snails and α-cobratoxins of snakes on the survival and proliferation of glioma C6 cells has been studied [43] to evaluate the presence and role of nAChR in C6 cells. It was found that α-conotoxins and α-cobratoxin contribute to the proliferation of glioma C6 cells.

The membranolytic activity of actinoporins, which are a pore-forming toxins produced by sea anemones, is also of great interest for the development of new antitumor drugs [97].

Although the development of vaccines and anticancer drugs are the two main areas of research in the production of recombinant toxins, there are also a number of other applications for which the production of recombinant toxins is of great interest. For example, killer toxins produced by yeast can be used in biological plant protection in order to obtain safe food products [98]. Moreover, killer toxins can also control postharvest pathogens [99].

Another example is the development of antidotes to these toxins for the treatment of poisoning caused by venomous bites of snakes, scorpions and spiders. The resulting antidotes consist of various antibodies, depending on the type of toxin against which they are created, and are injected. Poisonous bites of snakes, scorpions and spiders pose a serious threat and are the cause of high mortality rates worldwide. Despite the fact that antidotes based on polyclonal antibodies are the main means of saving people’s lives from poisonous animal bites, a wide variety of toxins in the composition of venoms of different species leads to the low effectiveness of such antidotes. It is worth nothing that such a thorough study of the properties of animal venoms (snakes, scorpions, spiders, bees, marine organisms) in the last few years made it possible to identify a number of compounds with antiviral activity in venoms. The possibility of use of their recombinant forms (e.g., recombinant peptide rEv37 from scorpion *Euscorpiops validus*, snake recombinant PLA2-CB isoforms) as the basis for antiviral drugs has been studied [100].

The outstanding therapeutic potential of BoNTs and TeNT made it possible to use them for the treatment of a wide range of neurological conditions related to hyperactivity in the periphery, acute and chronic muscle weakness. The potential to use the TeNT as a nanocarrier for neuron targeting and drug delivery to the central nervous system is another focus of research that is of great interest [101].

Thus, the purpose of obtaining recombinant toxins can be both the study of the properties of the toxin, the mechanisms of toxicity development in order to develop antidotes, the development of vaccines and the production of drugs based on the toxin, for example, to combat various tumor diseases and antimicrobials, which can also be used in biotechnological production.

## 4. Prediction of Toxicity of Synthetic Recombinant Proteins

**Figure 2:**
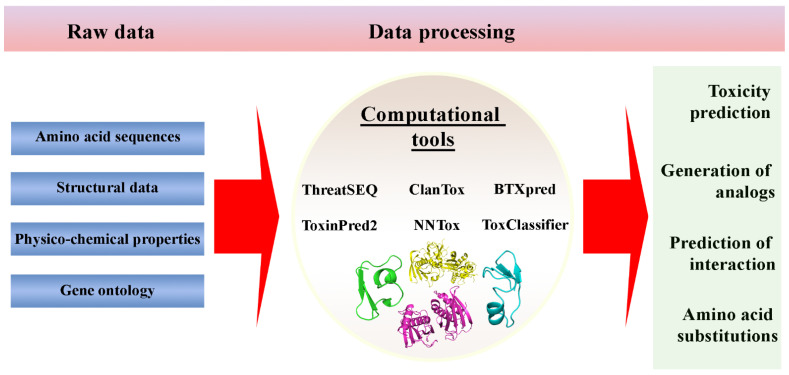
Machine-learning methods based tools for protein toxicity prediction.

The majority of the publications of recent years emphasize the importance of using bioinformatics methods to identify new variants of toxins and clarify the mechanisms of their toxic effects. Molecular modeling facilitates the understanding of the interaction of toxins with their receptors and/or targets, especially when these compounds are bound to the membrane, and biochemical approaches to the study of these processes are complex [102]. Due to advances in synthetic biology, the cost and time required for the development and synthesis of individual recombinant products are steadily decreasing. Many research laboratories regularly create genetically modified proteins as a part of their research activities. However, manipulations of amino acid sequences in proteins can lead to the unintended production of protein toxins. Therefore, the ability to determine the toxicity of a protein before its synthesis reduces the risk of the potential danger of synthetic production of protein toxins. For this purpose, various methods based on machine learning are being developed to predict the toxicity of proteins in silico based on a number of initial data (Figure 2).

ThreatSEQ, developed by Battelle Memorial Institute, identifies sequences of concern when compared with a database of known toxic proteins [103].

ToxinPred2 and other developed methods are aimed at detecting toxic bacterial proteins and peptides using machine learning methods based on information about the amino acid sequence [104,105,106].

ClanTox uses a machine learning method to classify known peptide inhibitors of ion channels [107], among which there are many well-characterized toxins.

ToxClassifier is available for use as a web application or as a separate downloaded tool for the purpose of classifying toxins from nontoxic protein sequences [108].

These methods are similar in that they use information about the amino acid sequence. However, new methods for predicting toxicity, including recombinant proteins, are emerging. In this vein, a new method of NNTox (prediction of protein toxicity based on a neural network) was recently presented [109], which can assess the potential toxicity of the sequence of the requested protein based on the annotation of the gene ontology (GO) of the protein [110]. GO is a controlled dictionary of protein functions that is widely used for annotation and prediction of functions. Earlier, the authors of the method developed a number of approaches to function prediction, including PFP and Phylo-PFP [111,112,113,114].

By combining genes or part of genes of similar or dissimilar proteins of various organisms, it is possible to obtain fusion proteins in vitro when designing recombinant proteins including toxins [18,25,42,54]. However real-time laboratory experiments to automatically predict the functionality of fusion proteins are expensive and time-consuming.

Today, a new method for predicting the functionality of fusion proteins based on a hybrid swarm algorithm of genetic optimization (HybGPSO) has been proposed to solve this problem [115]. The cellular component, the biological process and the molecular function of the unannotated fusion protein determined by the GO constitute the three main characteristics predicted by this algorithm.

Bacterial cells of *Bacillus thuringiensis* produce a natural three-domain Cry (3d-Cry) protein toxin (Bt), which is very actively used in different countries of the world as a bioinsecticide. The only existing tool for finding of Cry toxins, called BtToxin_scanner [116], has significant limitations, in particular, the limited size of the search query, insufficient accuracy and an outdated database.

In this regard, a fast and convenient CryProcessor tool has recently been developed, which enables productive and accurate finding of 3d-Cry toxins [117]. A unique feature of this tool is the ability to search for Cry toxin sequences directly on assembly graphs, which makes it possible to analyze raw sequencing data and overcome the problem of fragmented assemblies. Moreover, CryProcessor is able to accurately predict the location of domains in arbitrary sequences, allowing to extract sequences of specific domains beyond the limited number of toxins presented in CryGetter.

Thus, modern bioinformatic and genomic methods make it possible to assess the toxicity of recombinant proteins that are not related to known toxins and to discover new protein toxins in living organisms. These methods not only expand the diversity of toxins within known families but also reveal completely new classes of toxins, including artificially synthesized ones. One of the key problems associated with bioinformatically identified toxins is that there are few clues as to where to look for a possible mechanism of action beyond the information presented in the amino acid sequence and predicted domains.

## 5. Potential Enzymatic Antidotes for Recombinant Toxins

Due to the wide variety of toxins known to date and differences in the mechanisms of their action, there is an urgent need to create antidotes that both have a specific effect and are active against a wide range of toxins. The main directions of antidote development today are either the creation of various inhibitors capable of blocking the sites of binding of toxins to targets or the production of proteins (usually antibodies) capable of acting as bioscavengers via binding directly to the toxins themselves, thereby limiting their interactions with targets [118]. However, the search and development of new antidotes based on other principals, namely using molecules capable of detoxifying toxins by their enzymatic transformation into less toxic or nontoxic molecules, may become a promising alternative to existing solutions. To date, several enzymes are known that can act as antitoxins against various bacterial toxic substances, as well as enzymes that exhibit hydrolytic activity against PrP (Table 3, [119,120,121,122,123,124,125,126,127]).

The toxin/antitoxin (TA) systems are present in almost all strains of bacteria and archaea and consist of a toxin that slows down growth and an antitoxin, a compound that inhibits the activity of the toxin. Currently, there are six main classes of TA systems based on the nature of the antitoxin and the way in which the antitoxin inactivates the toxin [128].

It has recently been shown that there are at least three additional and different TA systems in which the antitoxin is an enzyme and the related toxin is a direct target of the antitoxin, and it has been proposed to use the type VII of TA system to designate those TA systems in which the enzyme-antitoxin can chemically modify the toxin post-translationally in order to neutralize it [129].

The Hha and TomB proteins from *E. coli* cells form an oxygen-dependent TA system in which the antitoxin oxidizes the Cys 18 of the toxin. It has been shown that Mob from *Yersinia* is an orthologue of TomB, and its only cysteine variant ^[C117S]^Mob can replace TomB as antitoxins in *E. coli* cells. Unlike other TA systems, ^[C117S]^YmoB temporarily interacts with Hha (rather than forming a stable complex) and enhances spontaneous oxidation of the conservative cysteine residue of Hha to -SOxH-containing compounds (sulfenic, sulfinic or sulfonic acid), which destabilizes the toxin [122]. In this case, oxidation of toxin molecules is used as a tool of its detoxification.

Recently, a study was published [119] in which the authors structurally and functionally characterized the putative TA locus Mtb Rv1044-Rv1045, demonstrating that it is a full-fledged TA system but uses a previously unknown mechanism of antitoxicity, including toxin phosphorylation. While Rv1045 encodes guanylyltransferaseTglT, which functions as a toxin, Rv1044 encodes a new atypical serine protein kinase TakA, which specifically phosphorylates a related toxin at the S78 residue, thereby neutralizing its toxicity.

The two-gene module encoding HEPN (the highest nucleotide-binding domain of eukaryotes and prokaryotes) and the related domain MNT (minimal nucleotidyltransferase) (HEPN/MNT) is considered the most common TA system in prokaryotes. However, its physiological function and the mechanism of neutralization remain unclear. Recently, it was discovered [120] that the MntA antitoxin (a protein with the MNT domain) acts as an adenylyltransferase and chemically modifies the HepT toxin (a protein with the HEPN domain) to block its toxicity as RNases. Biochemical and structural studies have shown that MntA mediates the transfer of three antimicrobial peptides (AMP) to a tyrosine residue near the HepT RNase domain in *Shewanella oneidensis*. In addition, in vitro enzymatic assays have shown that three AMP are transferred to HepT by MntA sequentially, with ATP serving as the substrate, and this polyadenylation is crucial for reducing the toxicity of HepT. In addition, the GSX10DXD motif, conserved among MntA proteins, is a key active motif for polyadenylation and neutralization of HepT. Thus, HepT/MntA is a new type of TA system, and the polyadenylation-dependent mechanism of TA neutralization prevails in bacteria and archaea.

Another example is the enzyme which is an antitoxin of the TA type V system that does not bind directly to the toxin but is able to cleave the mRNA of the corresponding toxin. Recently, ghoST has been studied for the first time as a type V of TA system that encodes a small toxin protein GhoT, which can damage the cell membrane, and antitoxin GhoS exhibited sequence-specific endoribonuclease activity with respect to the mRNA of the toxin GhoT [121].

The TA DarT/Dart system found in various bacteria, including the well-known pathogen *Mycobacterium tuberculosis*, has been identified and well characterized [124]. It turns out that the system toxin (DarT) is a domain of unknown function (DUFF) 4433, and the antitoxin (Dark) is a macrodomain protein. It has been found that DarT is an enzyme that specifically modifies thymidines on single-stranded DNA in a sequence-specific manner through a nucleotide-type modification called ADP-ribosylation. The authors found that this modification can be removed by DarG antitoxin by reversible ADP-ribosylation of DNA and suggested potential therapeutic benefits of such an enzyme–enzyme system in bacterial pathogens such as *M. tuberculosis*.

Prions can act as slow poisons, and therefore, these toxic proteins have the dangerous potential to be used as biological weapons [130]. Taking into account these facts, the search for enzymes that are capable of inactivating prions is an important current task of science. It is believed that the prion, which is the etiological agent of TSEs, consists of aggregated conformers of PrP (PrP^TSE^) rich in β-sheets obtained as a result of improper folding of the cellular form of the same protein. Enzymatic inactivation of prions in the absence of living microorganisms has been actively investigated in the last decade. A number of studies have reported successful enzymatic inactivation of prions, but many researchers have resorted to a combination of enzymatic treatment with high temperatures (>50 °C), high pH values (>9) [124], or both reaction conditions combined. The enzymatic inactivation of the prion under such extreme conditions limits the applicability of such treatment for use in the environment and in relation to most biological objects.

Proteases catalyze the hydrolysis of peptide bonds in the main chain of polypeptides and are therefore of particular interest because of their ability to inactivate prions. Despite the resistance of prions to proteolytic inactivation, a number of enzymes have been found that can cleave PrP^TSE^ and in some cases reduce the infectivity of prions. Most of the proteases found to date with the ability to cleave PrP^TSE^ are serine proteases. Subtilisin and subtilisin-like serine proteases are found in bacteria, archaea and fungi and represent one of the five superfamilies of prokaryotic serine proteases. Subtilisins are well represented among enzymes capable of hydrolyzing PrP^TSE^ [131].

The results of a study of the effectiveness of a commercially available subtilisin enzyme, Prionzyme, for the decomposition of soil-bound and unrelated PrP of CWD and hyper strain of transmissible mink encephalopathy (HY TME) depending on pH, temperature and treatment time are known [124]. The enzyme subtilisin efficiently decomposed PrP adsorbed on a wide range of soils and soil minerals below detection limits. Signal loss occurred rapidly at pH 12.5 and within 7 days under conditions typical of the natural environment (pH 7.4, 22 °C). There was no obvious difference in the efficiency of the enzyme action between bound and unbound CWD PrP. Thus, it was shown that, although adsorbed prions retain relative resistance to enzymatic cleavage compared to other brain homogenate proteins, they can be effectively decomposed when they are immobilized (in this case, when bound to soil). Topical application of a solution of the enzyme subtilisin can be an effective method of disinfection to limit the transmission of the disease through environmental “hot spots” of prion infectivity.

Two serine protease enzymes, subtilisin 309 and subtilisin 309-v, were used to treat brain homogenates containing a high level of prion infectivity under slightly alkaline conditions to study methods of prion decontamination. When confirming the elimination of the infectivity of abberantly folded rPrP, only one condition of hydrolysis (subtilisin 309 at 138 mg/mL, 55 °C, 14 h, pH 7.9) was considered statistically significant (*p* < 0.001) compared to the control [125].

Nattokinase (NK, also known as subtilisin NAT) is one of the most important extracellular enzymes produced by *Bacillus subtilisnatto* cells. The main interest in this enzyme is its fibrinolytic activity. The stability of this enzyme in the gastrointestinal tract makes it a useful agent for oral thrombolytic therapy. Thus, NK is used as a valuable food additive or as a nutraceutical. In addition to these valuable benefits, there are other uses attributed to NK, including the treatment of hypertension and Alzheimer’s disease. Additionally, it was found that NK is able to reduce the amyloid structure of recombinant human PrP fibrils [126]. In this regard, in the future, probably, it is possible to consider this enzyme as a candidate for the role of an antitoxin (antidote).

Keratinases are able to cleave the structure of β-keratin; thus, they can cleave PrP^Sc^, which consists of tightly packed β-sheets. The first keratinase detected for degradation of PrP^Sc^ was KerA from *B. licheniformis* PWD-1 cells [127].

An interesting study of PrP^TSE^ inactivation by lichens revealed that both aqueous and acetone extracts of three lichen species (*Parmeliasulcata*, *Cladoniarangiferina* and *Lobaria pulmonaria*) have the ability to decompose PrP-infected TSE hamsters, mice and deer. It has been found that PrP levels in PrP^TSE^-enriched preparations or infected brain homogenates also decrease after exposure to freshly harvested *P. sulcata* or aqueous lichen extract. Presumably, some other lichens may also have the potential to inactivate TSE infectivity in the landscape or be a source of agents for the decomposition of prions [131]; however, the active agent (biocatalyst) of this process has not yet been identified.

## 6. Conclusions

Different methods of isolation of recombinant proteins are used, depending on the source of the original toxin and the purposes for which it must be obtained. To date, *E. coli* is the most widely used expression system for the biosynthesis of recombinant toxins. At the same time, the very production of recombinant toxins occupies an important place in the study of the toxicity of various proteins/peptides, the mechanisms of their action and the disclosure of trends in the development of toxicity in vivo and helps researchers to look for new possible methods of their application in medicine and other fields of human activity. The main advantage of genes’ expression of recombinant forms of toxins is the possibility of producing these proteins/peptides in quantities sufficient to conduct experiments with them at the molecular and cellular levels. Moreover, modern methods of molecular biology and bioinformatics make it possible to carry out various genetic modifications of recombinant proteins and predict their toxicity and the most effective substitutions in their amino acid sequence.

## Figures and Tables

**Table 1:** Recombinant toxins from various origins and the purposes of their obtaining.

Protein	Origin	Reference
**Production**		
BoNT	Bacteria *Clostridium botulinum*	[11]
Killer toxins K1, K28, K1L	Yeast *Saccharomyces paradoxus*	[12,13,14]
Killer toxin Kpkt	Yeast *Tetrapisispora phaffii*	[15,16]
ppa1, Tppa2, Tce3, Cbi1	Scorpions of the genus *Tityus* and *Centruroides*	[17]
β/δ agatoxin-1	Spider *Agelena orientalis*	[18]
Purotoxin-1	Spiders of the genus *Geolycosa* sp.	[19]
Azemiopsin, Three-Finger Toxins	Viper *Azemipos feae*	[20,21]
MdumPLA_2_	Coral snake *Micrurus dumerilii*	[22]
APHC3, HCRG21	Sea anemone *Heteractis crispa*	[23,24]
**Toxicity assays**		
C3bot, C3bot_E174Q_, C2IIa	Bacteria *Clostridium botulinum*	[25,26,27]
LeTx	Bacteria *Bacillus anthracis*	[28]
HlyII	Bacteria *Bacillus cereus*	[29,30]
Cry1Ia	Bacteria *Bacillus thuringiensis*	[31]
BFT	Bacteria *Bacterioides fragilis*	[32]
EGFP-SbB, translocation domain (TD) of the diphtheria toxin	Bacteria *Corynebacterium diphtheriae*	[33,34]
In1B	Bacteria *Listeria monocytogenes*	[35]
LcrV	Bacteria *Yersinia pestis*	[36]
AtaT2	Bacteria *Escherichia coli*	[37]
Killer toxin Kpkt	Yeast *Tetrapisispora phaffii*	[38]
MeICT, KTx	Scorpion *Mesobuthus eupeus*	[39,40,41]
Tbo-IT2	Spider *Tibellus oblongus*	[42]
α-conotoxins, α-cobratoxin	Marine snail and snake venom	[43]
Three-Finger Toxins	Viper *Azemipos feae*	[44]
α-neurotoxins	Cobra *Naja melanoleuca*	[45]
Hct-S3	Sea anemone *Heteractis crispa*	[46]
**Immunology assays**		
BoNT	Bacteria *Clostridium botulinum*	[47]
Beta and epsilon toxins	Bacteria *Clostridium perfringens*	[48,49]
Cholera toxin subunit B (CTB)	Bacteria *Vibrio cholerae*	[50]
Ancrod, batroxobin, RVV-V	Snakes *Calloselasma rhodostoma*, *Bothrops atrox*, *Daboia russelii*	[51,52]
**Modifications**		
BoNT/B-MY, C2IN-C3lim	Bacteria *Clostridium botulinum*	[53,54]
DT389-YP7, s-DAB-IL-2(V6A), DT2219	Bacteria *Corynebacterium diphtheriae*	[55,56,57]
rPA83m + plant virus spherical particles (SPs)	Bacteria *Bacillus anthracis*	[58,59,60]
SElP + Zn	Bacteria *Staphylococcus aureus*	[61]
PE38 + AgNP	Bacteria *Pseudomonas aeruginosa*	[62]
CTB-KDEL	Bacteria *Vibrio cholerae*	[63]
GFP-L2-AgTx2	Scorpions *Mesobuthus eupeus* and *Orthochirus scrobiculosus*	[64]
LgRec1ALP1	Spiders of the genus *Loxosceles*	[65]
Ms 9a-1 fragments and homologues	Sea anemone *Metridium senile*	[66]

**Table 2:** Recombinant prion proteins and purposes of their biosynthesis and use.

Recombinant Protein	Purpose of Biosynthesis and Use	Reference
rPrP from a baculovirus-insect cell expression system (Bac-rPrP)	To determine whether Bac-rPrP^Sc^ is spontaneously produced in intermittent ultrasonic reactions	[71]
Mouse PrP (MoPrP, residues 89–230) in complex with a nanobody (Nb484)	To understand the role of the hydrophobic region in forming infectious prion at the molecular level	[72]
Transgenic mice expressing elk PrP (TgElk)	To test vaccine candidates against chronic wasting disease	[73]
rPrP from bank vole (BV rPrP)	To develop a method to dry and preserve the prion protein for long term storage	[74]
Murine rPrP	To study how RNA can influence the aggregation of the murine rPrP	[75]
Pr from the brains of clinically sick mice that had been intracerebrally inoculated with the Rocky Mountain Laboratory (RML) prion strain	To demonstrate that prions are not directly neurotoxic and that toxicity present in infected brain tissue can be distinguished from infectious prions	[76]
BV rPrP (amino acids 23–231)	To understand how different cofactors modulate prion strain generation and selection	[77]

**Table 3:** Enzymes as antidotes for toxic and prion proteins.

Protein	Enzyme	Mechanism of Action	Reference
Guanylyltransferase TglT from *Mycobacterium tuberculosis*	Serine protein kinase TakA	Specifically, phosphorylates the cognate toxin at residue S78, thereby neutralizing toxicity	[119]
HepT toxin from *Shewanella oneidensis*	Minimal nucleotidyltransferase (MNT)	MNT acts as an adenylyltransferase and mediates the transfer of three AMPs to a tyrosine residue next to the RNase domain of HepT	[120]
Bacterial GhoT toxin	Endoribonuclease	GhoS is a sequence-specific endoribonuclease that cleaves mRNA encoding GhoT, preventing its translation	[121]
Hha toxin from *Escherichia coli*	An oxygen-dependent antitoxin TomB	Inactivation of the Hha by oxidation with molecular oxygen mediated by the TomB	[122]
*Mycobacterium tuberculosis* toxin DarT	DarG—DNA ADP-ribosyl glycohydrolase	DarG could reverse the DNA ADP-ribosylation by DarT	[123]
PrP	Commercially available subtilisin enzyme, Prionzyme	Proteolytic inactivation/degradation	[124]
PrP	Subtilisin 309 and Subtilisin 309-v	Proteolytic inactivation/degradation	[125]
PrP	Nattokinase (NK, also known as subtilisin NAT) produced by *Bacillus subtilis natto*	NK is capable of decreasing amyloid structure of recombinant human PrP fibrils	[126]
PrP	Keratinase KerA from *B. licheniformis* PWD-1	Proteolytic inactivation/degradation	[127]

## Data Availability

Not applicable.
